# Does the Foot and Ankle Alignment Impact the Patellofemoral Pain Syndrome? A Systematic Review and Meta-Analysis

**DOI:** 10.3390/jcm11082245

**Published:** 2022-04-17

**Authors:** Nicolò Martinelli, Alberto Nicolò Bergamini, Arne Burssens, Filippo Toschi, Gino M. M. J. Kerkhoffs, Jan Victor, Valerio Sansone

**Affiliations:** 1 IRCCS Orthopedic Institute Galeazzi, Via Riccardo Galeazzi 4, 20161 Milan, Italy; n.martinelli@unicampus.it (N.M.); toschi.filippo@gmail.com (F.T.); valerio.sansone@unimi.it (V.S.); 2 Department of Biomedical, Surgical and Dental Sciences, University of Milan, Via Festa del Perdono 7, 20122 Milan, Italy; 3 Department of Human Structure and Repair, Ghent University Hospital, Corneel Heymanslaan 10, 9000 Ghent, Belgium; arne.burssens@ugent.be (A.B.); jan.victor@ugent.be (J.V.); 4 Department of Orthopedic Surgery, Amsterdam UMC, Academic Medical Center, Meibergdreeg 9, 1105 Amsterdam, The Netherlands; g.m.kerkhoffs@amsterdamumc.nl

**Keywords:** patellofemoral pain syndrome, hindfoot, rearfoot, alignment, kinematics, anterior knee pain

## Abstract

Background: A convincing association between the foot and ankle alignment (FAA) and patellofemoral pain syndrome (PFPS) remains debatable in the literature. Therefore, all studies investigating the role of FAA in patients with PFPS were systematically reviewed. Methods: A systematic literature search was performed on the databases PubMed, Embase, Cochrane Library, and Web of Science. Inclusion criteria were all studies investigating static and/or dynamic FAA factors and PFPS. Studies with less than 20 patients or with patellofemoral osteoarthritis were excluded. The quality assessment was based on Cochrane study criteria, and the maximum score was set at eight. Results: Of 2246 articles, only 13 case-control studies were eligible. Considering static FAA factors, two studies found an association with rearfoot eversion and one with rearfoot inversion. While examining dynamic FAA characteristics, one study found an association with rearfoot eversion range of motion and three with gait kinematics. No further associations were reported. The quality assessment mean score was 5.5 (SD = 0.97) corresponding to moderate quality. Conclusions: In contrast to our expectations, a limited number of studies were founded supporting an association between FAA and PFPS. At present, the quality of the literature is still poor and conflicting, thus the need for further studies to determine any association between FAA and PFPS.

## 1. Introduction

Patellofemoral pain syndrome (PFPS) is a relatively common disease of the lower limb in young and adult patients [1], especially in women [2,3] and sportsmen [4,5]. It is characterized by diffuse pain in/around the patella which worsens during prolonged sitting, squatting, kneeling, and stair climbing. Several intrinsic and extrinsic factors to the knee have been suggested, but no clear association has been reported for foot or ankle characteristics [6]. The literature seems to indicate Q-Angle, patellar characteristics, muscles strength, and activation time as the major risk factors [7,8], however, equivocal results have been found in the last two cited [9,10,11]. A previous review found static and dynamic foot parameters associated with PFPS [12], but no strong clinical evidence confirmed its validity [13]. In the complexity of rehabilitation plans, this lack of evidence does not fit completely with the everyday treatments used, such as personalized or prefabricated orthosis [14], thus resulting in discordant clinical long-term outcomes [15,16]. Moreover, it has been examined [17] how rehabilitative exercises focused on foot stability and taping significantly contribute to the improvement of pain. Recently, it has been pointed out [7] to rethink the need to approach PFPS not only with strength training but also with reprogramming exercises of acquired movements.

Hence the need for further investigation on the etiology of PFPS is evident. For this reason, this systematic review aims to summarize static and kinematic characteristics of the foot related to PFPS. It was hypothesized that the PFPS is impacted by different static and kinematic factors related to the foot and ankle alignment.

## 2. Materials and Methods

The original protocol for this study was registered on PROSPERO, the international prospective register of systematic reviews, which can be accessed online (CRD42021236739). The Database of Abstracts of Reviews of Effects, the Cochrane Database of Systematic Reviews, and PROSPERO could not identify previously performed reviews investigating static and kinematic factors of the foot and ankle alignment (FAA) related to the PFPS. A systematic review of the literature was conducted in accordance with the Preferred Reporting Items for Systematic Reviews and Meta-Analyses (PRISMA) guidelines. The following electronic databases were consulted: Medline (Pubmed), Embase (Ovid), Web of Science, and Cumulative Index to Nursing and Allied Health Literatura (CINAHL).

### 2.1. Inclusion Criteria

All randomized control trials (RCTs) and case-control studies examining at least one foot/ankle risk factor (static or dynamic) associated with PFPS published in English or French were included, published between January 1990 and 1 April 2020. No sex limitations were applied, otherwise, the age limit was set between 10 and 40 years. Due to the lack of consistent terminology for PFPS, all definitions for PFPS and its synonyms were accepted. Patients with chondromalacia patella were included if the authors intended chondromalacia patella to be a description of PFPS.

### 2.2. Exclusion Criteria

Studies with less than 20 subjects were excluded. Other criteria consisted of previous operative treatment or arthroscopy, other secondary knee-related problems (bursitis, tendinopathy, osteochondritis, neuromas, intraarticular pathology (such as osteoarthritis), plica syndrome, tumor, and rheumatologic diseases, and more rare pathologies) and concomitant use of orthotics, arch supports, and night splints. Excluded from the analysis were also articles involving a treatment intervention.

Two reviewers (A.N.B., N.M.) worked on the selection of cases independently according to inclusion and exclusion criteria. For the selected references, a final decision about inclusion was made based on the full-text articles. These articles were reviewed independently. If there was a disagreement, the criteria for inclusion were discussed until a consensus was reached. A third review team member (A.B.) had been consulted if an agreement could not be reached.

### 2.3. Search Strategy

The following electronic databases were consulted for the primary research: Pubmed (Medline), Embase (Ovid), Web of Science, and CINAHL (EBSCO). Identical search strategies were performed on all databases combining the following keywords: (rearfoot OR hindfoot OR foot OR ankle) AND (arthralgia OR knee joint OR anterior knee pain) OR (patell* OR femoropatell* OR femoropatell* OR retropatell*) AND (pain OR syndrome OR dysfunction) AND (risk factor OR association OR relative risk OR odds ratio). Articles not published in English or French were excluded, and the research was filtered to the period from January 1990 to April 2020. References of included studies and reviews on PFPS were screened for further citations (Appendix A).

### 2.4. Quality Assessment

Methodological quality assessment of each study was performed by two reviewers (N.M., A.N.B.) independently using a quality assessment in accordance with the Dutch Cochrane Centre and modified by Lankhorst et al. [12].

The list consists of eight items (Table 1). The quality of the studies was assessed by scoring each of the study criteria as ‘positive’, ‘unclear’, or ‘negative’. Positive criteria were scored with one point. Disagreements were solved by discussion. Percent agreement was calculated to determine the agreement between the two reviewers. The quality score of each study was measured by summing up the total number of positive criteria. The quality of the evidence was categorized: ‘very low’: 0 to 1 points; ‘low’: 2 to 4 points; ‘moderate’: 5 to 6 points; ‘excellent’: 7 to 8 points.

### 2.5. Data Extraction

One reviewer extracted relevant data from the publications. Information on study design (type of study, author, and year of publication), study population (number of cases/controls enrolled and analyzed), group characteristics (gender, age, and definition of PFPS), and assessment method were extracted using a standardized form. Means and standard deviation (SD) were extracted for variables of interest, which included (but were not limited to) demographics (such as sex, body mass index (BMI)), foot/ankle characteristics (i.e., static alignment or dynamic function). When possible, the mean differences (MDs) with SD were extracted or calculated from the original studies. Other comments that could not be matched within any of the items described above and were judged to be possibly important for this review were noted.

### 2.6. Statistical Analysis

A meta-analysis was performed on foot and ankle characteristics, that had a consistent definition and assessment across different studies, to obtain a pooled estimate of the size of the risk factor. Meta-analysis was performed using Review Manager Software version 5.3 (RevMan v5.3, The Nordic Cochrane Centre, Copenhagen, Denmark). Heterogeneity between studies was calculated using χ^2^ and I^2^ tests, a random-effects model was used for the Forest plot.

## 3. Results

**Figure 1:**
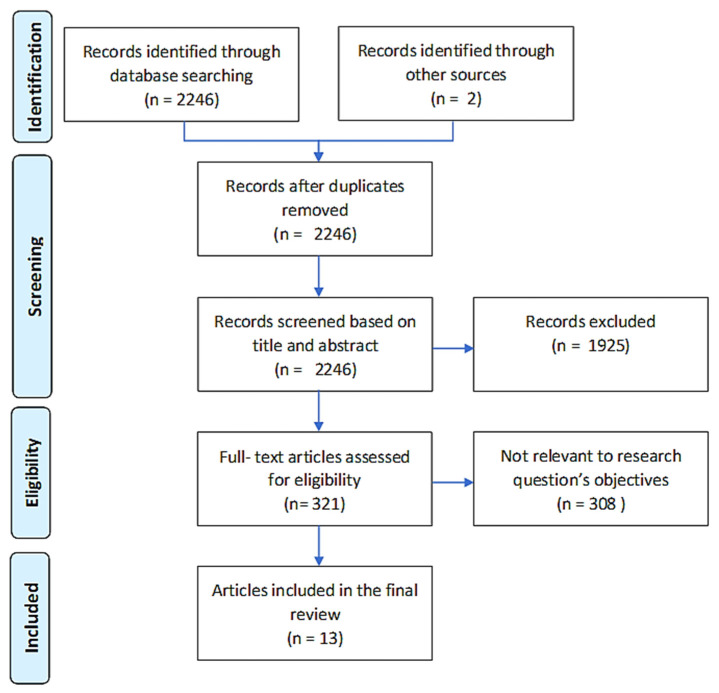
Flow chart.

A total of 2246 relevant articles were identified and screened for eligibility. Two studies were ruled out for multiple publications with identical data made by the same author [2,15]. From titles and abstracts, 321 articles were selected for a full-text review, among them, 11 fulfilled inclusion criteria. The reviewers extracted two more studies from article references [18,19], totaling 13 studies [3,18,19,20,21,22,23,24,25,26,27,28,29] at the end of the screening (Figure 1).

The quality assessment was performed by two authors which agreed on 91% of the quality items (95 over 104 items) (Table 1). The quality score ranged from 4 to 7 and the mean quality score for the studies was 5.5 ± 0.96. Only three studies included more than 50 patients per group, while only two did not compare homogeneous groups. All the studies provided a clear definition of the inclusion and exclusion criteria and the measured outcomes. In only one study the observer was blinded to the health status (PFPS versus controls) of the subjects.

### 3.1. Static Alignment Measures

Foot and ankle characteristics involving the static alignment were reported in eight studies [3,19,20,21,22,23,24,25,29] (Table 2 and Table 3). In these studies, the most investigated characteristic was the rearfoot position, three of them found an association between static alignment and PFPS [3,25,29]. Two studies [3,25] found a positive correlation; Barton et al. [25] reported that PFPS subjects had a more pronated foot posture and greater foot mobility than the control group using the subtalar joint neutral position as a reference (5.8° ± 3.3° vs. 2.8° ± 3.6°, *p*-value < 0.05), and Novello et al. [3] reported a greater foot posture index in PFPS subjects than healthy subjects (7.0 vs. 5.0, *p*-value 0.003). On the contrary, one study found a negative correlation: Steinberg et al. [29] described a varus rearfoot was significantly more frequent in PFPS young dancers than in the healthy control group (pes varus 8.9%-pes valgus 3.8%). The remaining five studies could not demonstrate any association. Linvingston et al. [22], comparing left and right rearfoot eversion angle (REA) in asymptomatic, unilateral, and bilateral knee pain, and Thomeé et al. [19], measuring the angles between lower leg, calcaneus, and horizontal axis, found no correlation between hindfoot alignment and PFPS.

**Figure 2:**
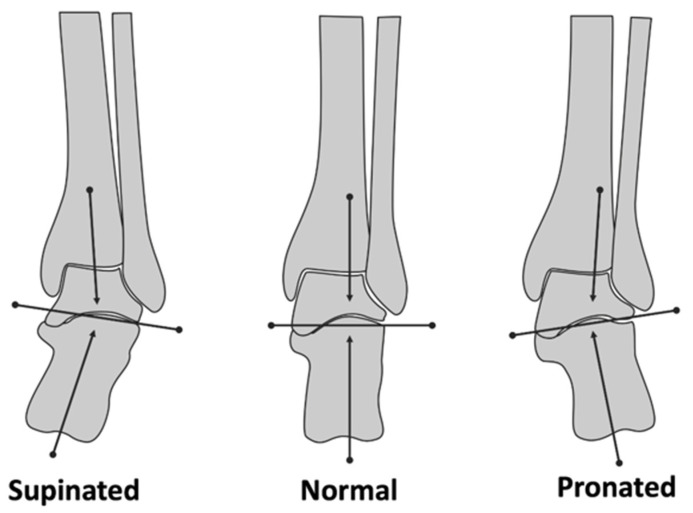
Differences in foot and ankle alignment.

Barton et al. [25] and Thomeé et al. [19] measured REA in bipedal relaxed position: the first used a digital inclinometer taking as reference the perpendicular line to the floor, the second [19] adopted a standard goniometer on a video recording after having placed some signs as landmarks on the lower limb to identify the axis of the calcaneus and the tibia. Again, Barton [25] made the same measures in the non-weight-bearing stance with the subtalar joint in the neutral position. Additionally, Linvingston et al. [22] and Novello et al. [3] took as references the axis of the calcaneus and the axis of the tibia in a weight-bearing position, Steinberg et al. [29] assessed hind-foot alignment valgus/varus in standing anatomical position without goniometric measuring, referring to the evaluation made by Magee and Manske [30] (Figure 2).

Duffey et al. [21] reported a significantly lower arch index in PFPS group compared to the normal group (0.238 vs. 0.251, *p*-value < 0.05), while no association was observed between arch height indexes and PFPS by Dierks et al. and Thomée et al. [19,24]. Two studies [19,21] reported arch index as the ratio of the area of the middle third of a footprint relative to the total area excluding the toes. Dierks et al. [24] measured arch height index as the ratio of the dorsum height (at 50%-foot length), divided by the truncated foot length.

No significant difference was reported between the prevalence of pes cavus/flat foot in PFPS patients and control subjects in the study of Haim et al. [23].

### 3.2. Dynamic Alignment Measures

Eight studies [3,18,20,21,24,26,27,28] examined kinematic variables; five [18,20,21,24,28] used linear gait analysis, three of them [18,21,24] during running and two [20,28] while walking, and three adopted stars descending [3,27], or ascending [26], dynamic studies (Table 2 and Table 3).

**Figure 3:**
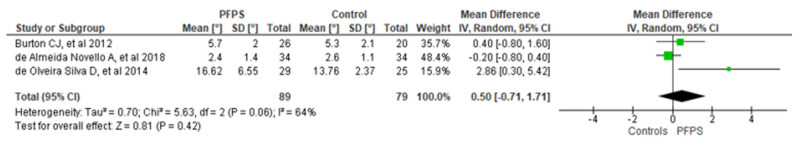
Forest plot.

The rearfoot eversion range of motion (ROM) was the most measured variable [3,18,26,28] (Figure 2). De Oliveira Silva et al. [26] found a significantly higher ROM in PFPS than the control group (16.66 ± 6.55 vs. 13.76 ± 2.37), while Barton et al. [28] and Novello et al. [3] found no difference. No association was found after pooling (MD 0.50; 95% CI −0.70 to 1.71) (Figure 3). Moreover, Luz et al. [18], and Barton et al. [28] found an association between peak rearfoot eversion and peak tibial internal rotation and peak femur adduction, greater in the first group than in the second, but no difference in kinematic between PFPS and control group.

One study [27] recorded a larger contact area of medial rearfoot and midfoot and an overall lower peak pressure in patients with PFPS compared to controls when descending stairs. The same author [20] reported in a following article a greater contact area over the medial and central rearfoot during heel strike, and on the forefoot during midstance and higher pressure on the lateral forefoot in the propulsion phase in comparison to the control group.

Duffey et al. [21] observed lower pronation speed in patients with PFPS compared to controls. Furthermore, patients with PFPS also had a significant reduction in calcaneus-tibia touchdown angle (MD 2.80 ± 10.9) and foot pronation angle during the first 10% of the stance while running (MD −1.30 ± 4.5). De Oliveira Silva et al. [26] supported the reliability and differentiation capability of the rear foot ROM measure during climbing stairs, while static parameters were of no use, on the other hand, according to Novello et al. [3], kinematic changes while descending stairs should be used with caution in the evaluation and decision process, as they have a low discriminatory capacity in identifying a subject with PFPS and one not.

## 4. Discussion

In this systematic review, we have not found a clear relationship between PFPS and foot and ankle alignment. Marked discrepancies were found in the methodologies, results, and conclusions in the studies analyzed. Furthermore, the lack of studies, the small sample size, and the moderate quality of the protocols evoke the need for further studies containing a higher level of evidence to determine whether alteration in foot alignment can contribute to the development, progression, and treatment of PFPS.

Static and dynamic factors assessing the foot and ankle alignment are often considered one of the risk factors for PFPS [1,31]. However, the principal finding of this systematic review found conflicting evidence on the association between the foot and ankle alignment and PFPS. This discordance between studies could be attributed to differences in recording, tests used (ascending descending stairs, running, walking), subjects (soldiers, dancers, recreational runners), and measurement methods. Moreover, these different methodologies limited pooling for all the variables analyzed in this review due to the lack of standardization in the different protocols. Furthermore, only case-control studies were eligible for this review according to inclusion and exclusion criteria. Based on the quality assessment used, studies had a mean score of 5.5 out of 8, and it was remarkable that only 3 of 13 studies had more than 50 patients per group, and just in one, the outcome assessor was blinded.

This study identified a general agreement that static foot parameters are poorly correlated to PFPS. Three studies [19,22,23] found a lack of correlation between hindfoot alignment and PFPS, while Barton et al. [25], Novello et al. [3], and Steinberg et al. [29] reported conflicting results. The first two found a more pronated position, while the last a cavus foot posture and greater foot-joint mobility in PFPS patients than in the control group.

The arch index was a static variable assessed in different studies. In contrast with a previous systematic review [12], we decided not to pool data regarding arch index [19,21,24] since only two articles provided numeric data with similar standardized measurements [21,24]. However, the association between the arch index and PFPS was controversial, only Duffey et al. [21] found a possible link.

Kinematic studies showed a weak association between dynamic variables of the foot and PFPS, with a discrepancy in the outcomes. Only the variable rearfoot eversion ROM was possible to pool due to the same measure methodology [3,26,28]. The pooled data of the three studies showed no difference between PFPS patients and the control group, but statistical heterogeneity was large (I^2^ = 68%). These findings may be explicated by dissimilar dynamic analysis: Barton et al. [28] used a walking linear gait analysis, Novello et al. [3] a descending stair, and de Oliveira Silva et al. [26] an ascending stair model.

**Figure 4:**
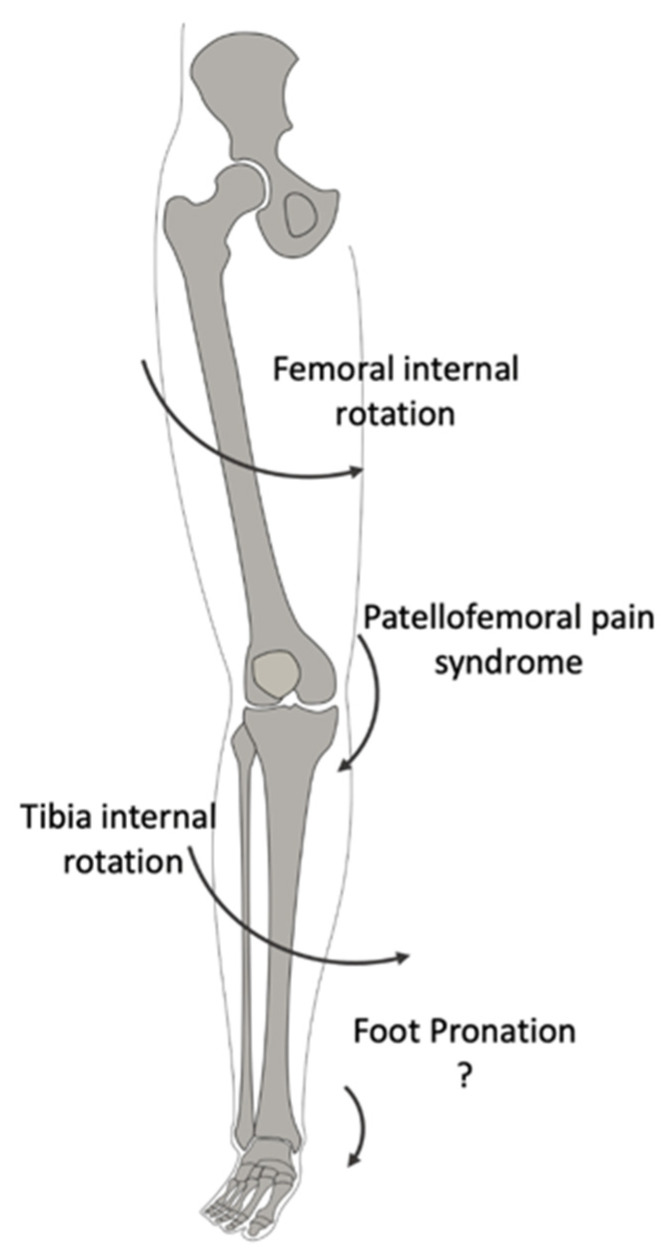
Lower Limb alignment. This sign (?) has been inserted because the figure is still a theorical explanation of the association between the two phenomenons.

Barton et al. [28] and Luz et al. [18] highlighted the association between peak rearfoot eversion, peak tibial internal rotation, and peak femur adduction, while de Oliveira Silva et al. [26] reported greater rearfoot eversion ROM in PFPS patients than the control group. In addition, Aliberti et al. [20] reported an increased medial-to-lateral roll-over of the foot during gait. These data may support a previous belief that prolonged or excessive foot pronation [32] does not permit the tibia to externally rotate during extension of the limb, thus leading to a compensatory excessive internal rotation of the femur. This movement lateralizes the patella, thus causing its mal-tracking. Based on this assumption, the excessive pronation of the subtalar joint was historically identified as a risk factor for a dysfunctional biomechanical mechanism of the lower limb, leading to kinematics adaptation of the other joints (Figure 4).

Duffey et al. [21] found no difference in rearfoot eversion ROM, but lower pronation and greater stiffness of the foot during the heel strike in subjects with PFPS. They stated that this abnormal kinematics does not allow the ground contact forces to be adequately dissipated, generating stress on the entire lower limb and therefore also on the knee. However, as aforementioned, the clinical evidence of this theory is fluctuating and not significant. Additionally, this review supports the lack of clinical association between foot characteristics and patellar femoral pain [8,9,12].

This study encountered several important limitations. Firstly, in common with other systematic reviews, some papers may not have been identified with the search criteria that we used. This could be attributed to the initial search terms, which considered a period from 1990 to 2020, thus excluding all the articles published before, or exclusion of papers not written in English or French. However, additional screening of the references was performed to improve the process and two additional studies were identified. Secondly, only a relatively small number and heterogenous group of studies were found to be eligible for this systematic review. There was considerable variation in the static and kinematic outcomes reported, thus not permitting us to perform a meta-analysis or calculate an overall effect of the foot and ankle alignment on the patellofemoral pain syndrome. Future research should standardize the recording of investigational methodologies and radiological measurements. Thirdly, only case-control studies were eligible based on inclusion and exclusion criteria. The quality assessment showed that these studies had a relatively medium quality, only three had an adequate sample size and just one was blinded. Thus, future studies should use prospective cohorts with a larger number of patients. Methodological difficulties with current PROMs could be addressed by using newer patient-reported outcome measures and information systems (PROMIS). Similarly, the shortcomings, such as rotational errors and the superposition of osseous hindfoot structures, in quantifying static foot and ankle alignment using plain radiography can be overcome by the recent availability of weight-bearing cone-beam CT imaging, which is able to take an image of the foot to the knee [33,34,35,36,37,38]. Standardization differences in the kinematic analysis can be overcome by newer methodologies such as dual fluoroscopy [39,40] or 4D CT analysis [41,42].

## 5. Conclusions

Marked discrepancies were found in the methodologies, results, and conclusions in the studies analyzed in this systematic review. Conflicting evidence was noticed regarding the association between PFPS and deformity of the foot and ankle. Furthermore, the lack of prospective studies, the small sample size, and the moderate quality of the protocols evoke the need for further studies containing a higher level of evidence to determine whether alteration in the foot alignment can contribute to the development, progression, or treatment of PFPS.

## Figures and Tables

**Table 1:** Quality assessment.

Author & Publication Year	1	2	3	4	5	6	7	8	Total Number
Thomee’ et al., 1995	1	0	1	1	1	1	0	?	6
Duffey et al., 2000	0	1	1	1	1	1	1	?	6
Livingston et al., 2003	1	0	0	1	1	1	0	?	4
Haim et al., 2006	0	1	0	1	1	1	?	0	4
Dierks et al., 2008	1	0	1	1	1	1	1	?	6
Barton et al., 2010	1	0	1	1	1	1	1	1	7
Aliberti et al., 2010	1	0	1	1	1	1	1	0	6
Aliberti et al., 2011	1	0	1	1	1	0	?	?	4
Barton et al., 2012	1	0	1	1	1	1	1	?	6
De Oliveira Silva et al., 2014	1	0	1	1	1	1	1	0	6
Steinberg et al., 2017	1	1	1	1	1	1	0	?	6
Novello et al., 2018	1	0	1	1	1	1	?	?	5
Luz et al., 2018	1	0	1	1	1	1	?	?	5

Legend: (1) Open populations study groups or recruited from primary and secondary care or only women recruited; (2) number of cases >50 for each group; (3) study groups comparable for age and gender; (4) clear definition of exclusion and inclusion criteria was described; (5) clear definition of outcome measure was described; (6) risk estimates were presented or raw data were given that allow the calculation of risk estimates; (7) methods used for confounding was described; (8) blinding of outcome assessor on health status subjects. Scores: (1) positive (0) negative (?) unclear.

**Table 2:** Group general characteristics.

Author & Publication Year	Case Group				Control Group			
	N	Age	Height (cm)	Weight (Kg)	N	Age	Height (cm)	Weight (kg)
Thomee’ et al., 1995	40	20 ± 3	169 ± 6	64 ± 9	20	22 ± 3	168 ± 6	61 ± 9
Duffey et al., 2000	99	36 ± 9.9 &	172.1 ± 10.9 & *	69.5 ± 13.9 & *	70	35 ± 8.4 &	174.5 ± 9.2 & *	70.2 ± 10.9 & *
Livingston et al., 2003	25	27.1 ± 7.9 $	174.3 ± 7.2 $	74.4 ± 10.3 $	50	26 ± 7 $	171.7 ± 5.5 $	74. 3 ± 11.5 $
Haim et al., 2006	61	19.4 ± 1.2	/	/	25	24.1 ± 6.5	/	/
Dierks et al., 2008	20	24.1 ± 7.4	171 ± 10	65.8 ± 12.6	20	22.7 ± 5.6	170 ± 8	63 ± 9.2
Barton et al., 2010	20	22.8 ± 4.1	167.9 ± 6.8	66.8 ± 11.3	20	21.9 ± 3.5	169.9 ± 8.3	63.9 ± 14.0
Aliberti et al., 2010	30	30 ± 7	165 ± 9	63 ± 11	44	30 ± 8	165 ± 8	60 ± 11
Aliberti et al., 2011	22	30 ± 7	165 ± 9	63 ± 12	35	29 ± 7	164 ± 8	60 ± 11
Barton et al., 2012	26	25 ± 5	169 ± 9	67 ± 14	20	23 ± 2	171 ± 8	66 ± 15
De Oliveira Silva et al., 2014	29	21.9 ± 2.7	165 ± 5	65.7 ± 10.8	25	22.1 ± 3.7	165 ± 4	62.3 ± 7.3
Steinberg et al., 2017	34	10–11	140.4 ± 8.4	31.1 ± 5.3	34	10–11	140.2 ± 8.2	31 ± 5.2
	120	12–14	154.5 ± 8.1	43.1 ± 7.7	120	12–14	154.9 ± 8.3	43.5 ± 7.8
	117	15–16	160.6 ± 5.1	49.8 ± 5.6	117	15–16	161 ± 5.4	50.2 ± 5.5
Novello et al., 2018	34	23 (20–31) ^	1.61 (1.6–1.7) ^ *	58 (52–62) ^	34	26 (23–28) ^	1.60 (1.55–1.65) ^ *	55 (51–61) ^
Luz et al., 2018	27	27 ± 4.2	172 ± 0.01	71.2 ± 12.8	27	26 ± 5.6	174 ± 0.1	72.5 ± 14.1

& Calculated using formula: SD = SEM ∗ √n. $ extracted from data in the article. ^ IQR = interquartile range. * *p*-value < 0.05.

**Table 3:** Variables analyzed.

**(Part 1/3)**		
**Author & Publication Year**	**Variables**	**Mean Difference (Patients–Controls) ± SD**
Thomee’ et al., 1995	-Angle between lower leg and horizontal	0.1 ± 3.8
	-Angle between calcaneus and horizontal	0.7 ± 3.5
	-Angle between lower leg and calcaneus	1.1 ± 4.5
	-Arch index	0.5 ± 10.2
Duffey et al., 2000	-Dorsiflexion ankle ROM (°)	0.4 ± 8.4 &
	-Plantarflexion ankle ROM (°)	1.1 ± 9.9 &
	-Arch index	0.013 ± 0.762 & *
	-Calcaneus-tibia touchdown angle (°)	2.8 ± 10.9 &
	-Pronation through first 10% of stance (°)	−1.3 ± 4.5 & *
	-Maximum pronation (°)	0.5 ± 8.4 &
	-Total pronation (°)	−1.5 ± 8.4 &
	-Calcaneus-vertical tibial-distal angle (°)	2 ± 10.3 &
	-Time to maximum pronation (%stance)	1.4 ± 18.0 &
	-Time to maximum eversion (%stance)	1.2 ± 14.5 &
	-Initial pronation velocity (° s^−1^)	−70 ± 231.0 &
	-Maximum pronation velocity (° s^−1^)	−79 ± 237.1 &
	-Time to maximum pronation velocity (%stance)	1.5 ± 9.0 &
Livingston et al., 2003	-Right rearfoot angle (°)	−1.5 ± 6.9
	-Left rearfoot angle (°)	−0.5 ± 7.2
Haim et al., 2006	-Pes cavus (patients vs. controls)	16% vs. 16%
	-Pes planus (patients vs. controls)	31% vs. 15%
Dierks et al., 2008	-Arch height index	0.011 ± 0.036
	-Rearfoot angle during cinematic study	Not disponible
Barton et al., 2010	Relax stance	
	-Longitudinal arch angle (°)	−6.8 ± 10.5 *
	-Foot posture index (°)	2.4 ± 4.9 *
	-Normalized vertical navicular height (%foot length)	−2 ± 4.3
	-Calcaneal Angle (°)	1.8 ± 5.5
	-Normalized dorsal arch height (%foot length)	−1 ± 3.0
	Foot posture relative, subtalar joint neutral	
	-Normalized navicular drop (%foot length)	1.6 ± 2.3 *
**(part 2/3)**		
**Author & Publication Year**	**Variables**	**Mean Difference (Patients–Controls) ± SD**
Barton et al., 2010 (continue)	Foot posture relative, subtalar joint neutral	
	-Normalized dorsal arch height difference (%foot length)	0.7 ± 1.0 *
	-Normalized navicular drift (%foot length)	1.6 ± 2.4 *
	-Longitudinal arch angle difference (°)	3 ± 4.7 *
	-Calcaneal Angle difference	2.6 ± 4.8 *
	Sagittal plane measures	
	-First metatarsophalangeal joint (°)	3.1 ± 18.6
	-Ankle dorsiflexion, knee flexed (°)	3.8 ± 9.5
	-Ankle dorsiflexion, knee extended (°)	2.5 ± 10.4
Aliberti et al., 2010	Contact area (cm^2^)	
	-Medial Rearfoot	1.8 ± 5.3
	-Central Rearfoot	0.1 ± 3.3
	-Lateral Rearfoot	−0.4 ± 6.6
	-Mid-foot	3.6 ± 7.6
	-Medial forefoot	1.2 ± 4.0
	-Lateral forefoot	1.2 ± 3.8
	Pressure-time integra (kPa·s)	
	-Medial Rearfoot	3.5 ± 25.5
	-Central Rearfoot	−0.6 ± 27.9
	-Lateral Rearfoot	−0.7 ± 30.1
	-Mid-foot	0.7 ± 18.9
	-Medial forefoot	−10.7 ± 46.3
	-Lateral forefoot	−5.5 ± 47.2
Aliberti et al., 2011	-Contact area medial rearfoot (kPa·s) -Contact area central rearfoot (kPa·s) -Contact area lateral rearfoot (kPa·s) -Contact area midfoot (kPa·s) -Contact area medial forefoot (kPa·s) -Contact area lateral forefoot (kPa·s) -Peak pressure medial rearfoot (kPa·s) -Peak pressure central rearfoot (kPa·s) -Peak pressure lateral rearfoot (kPa·s) -Peak pressure midfoot (kPa·s) -Peak pressure medial forefoot (kPa·s) -Peak pressure lateral forefoot (kPa·s)	Not disponible
**(part 2/3)**		
**Author & Publication Year**	**Variables**	**Mean Difference (Patients–Controls) ± SD**
Barton et al., 2012	-Gait velocity (m/s)	−0.1 ± 0.2
	Peak angles (°)	
	-Rearfoot eversion	1.6 ± 5.4
	Range of motion (°)	
	-Rearfoot eversion	0.4 ± 2.9
De Oliveira Silva et al., 2014	-Rearfoot eversion ROM (°)	2.9 ± 5 *
	-Rearfoot static angle (°)	1.7 ± 5.2
Steinberg et al., 2017	-Hind-foot varum (patients vs. controls)	17.4% vs. 8.5% *
	-Hind-foot valgus (patients vs. controls)	17.5% vs. 13.7%
	-Ankle plantar-flexion (°)	−2.16 ± 10.54 *
	-Ankle dorsiflexion (°)	1.37 ± 6.48 *
Novello et al., 2018	-Foot posture index	5 (3–6) vs. 7.0 (5–8) ^ *
	Hindfoot in relation to the horizontal plain (ROM)	
	-(+) Dorsiflexion (−) Plantar flexion	−1.0 ± 3.9 *
	-(+) Inversion (−) Eversion	−0.2 ± 1.8
	-(+) Internal (−) External rotation	−0.3 ± 3.7
	Hindfoot in relation to the tibia (ROM)	
	-(+) Dorsiflexion (−) Plantar flexion	−1.9 ± 7.2 *
	-(+) Inversion (−) Eversion	−1.4 ± 7.1 *
	-(+) Internal (−)External rotation	−1.7 ± 4.7 *
	Forefoot in relation to the hindfoot (ROM)	
	-(+) Dorsiflexion (−) Plantar flexion	1.9 ± 5.6 *
	-(+) supination (−) Pronation	1.3 ± 3.2 *
	-(+) Adduction (−) Abduction	−1 ± 2.6 *
Luz et al., 2018	Peak angles	
	-Rearfoot eversion	0.47 ± 5.7
	Range of motion	
	-Rearfoot eversion	0.89 ± 5.8

& Calculated using formula: SD = SEM ∗ √n. ^ data expressed as median (interquartile interval). * *p*-value < 0.05.

## Data Availability

Not applicable.

## Appendix A

**Table A1:** Database and search chain used.

Database	Search Chain
Pubmed	(hindfoot*[tw] OR rearfoot*[tw] OR foot*[tw] OR ankle*[tw] OR forefoot*[tw]) AND (patellofemoral pain*[tw] OR patellofemoral syndrom*[tw] OR patello-femoral pain*[tw] OR patello-femoral syndrom*[tw] OR anterior knee pain*[tw] OR patellofemoral disorder*[tw] OR patello-femoral disorder*[tw]) OR ((arthralg*[tw] OR pain*[tw]) AND (knee joint[mesh] OR knee*[tw] OR patell*[tw] OR femoropatell*[tw] OR femoro-patell*[tw] OR retropatell*[tw] OR retro-patell*[tw] OR lateral facet*[tw] OR lateral compr*[tw] OR lateral press*[tw] OR odd facet*[tw] OR genu[tw]) AND (syndrom*[tw] OR dysfunct*[tw] OR disorder*[tw] OR chondromal*[tw] OR chondropath*[tw]))) AND (associat*[tw] OR risk*[tw] OR probabil*[tw] OR odds*[tw] OR relat*[tw] OR prevalen*[tw] OR predict*[tw] OR caus*[tw] OR etiol*[tw] OR interact*[tw]
Embase	((‘rearfoot’/exp OR rearfoot OR ‘hindfoot’/exp OR hindfoot OR ‘foot’/exp OR foot OR ‘ankle’/exp OR ankle) AND (‘arthralgia’/exp OR arthralgia OR ‘knee joint’/exp OR ‘knee joint’ OR ((‘knee’/exp OR knee) AND (‘joint’/exp OR joint)) OR ‘anterior knee pain’/exp OR ‘anterior knee pain’ OR (anterior AND (‘knee’/exp OR knee) AND (‘pain’/exp OR pain))) OR patell* OR femoropatell* OR retropatell*) AND (‘pain’/exp OR pain OR ‘syndrome’/exp OR syndrome OR dysfunction) AND (‘risk factor’/exp OR ‘risk factor’ OR ((‘risk’/exp OR risk) AND factor) OR ‘association’/exp OR association OR ‘relative risk’/exp OR ‘relative risk’ OR ((‘relative’/exp OR relative) AND (‘risk’/exp OR risk)) OR ‘odds ratio’/exp OR ‘odds ratio’ OR (odds AND (‘ratio’/exp OR ratio)))
Web of Science	(((hindfoot* OR rearfoot* OR foot* OR ankle* OR forefoot*) AND (patellofemoral OR “patello-femoral” OR “anterior knee”) AND (pain* OR syndrom* OR disorder*)) OR ((arthralg* OR pain*) AND (knee* OR patell* OR femoropatell* OR retropatell* OR “retro-patellar” OR “lateral facet” OR “lateral compression” OR “lateral pressure” OR “odd facet” OR genu) AND (syndrom* OR dysfunct* OR disorder* OR chondromal* OR chondropath*))) AND (associat* OR risk* OR probabil* OR odds* OR relat* OR prevalen* OR predict* OR caus* OR etiol* OR interact*)
CINAHL	(rearfoot OR hindfoot OR foot OR ankle) AND (arthralgia OR knee joint OR anterior knee pain) OR (patell* OR femoropatell* OR femoropatell* OR retropatell*) AND (pain OR syndrome OR dysfunction) AND (risk factor OR association OR relative risk OR odds ratio).
